# Analysis of the Gene Networks and Pathways Correlated with Tissue Differentiation in Prostate Cancer

**DOI:** 10.3390/ijms25073626

**Published:** 2024-03-24

**Authors:** Alexandru Filippi, Justin Aurelian, Maria-Magdalena Mocanu

**Affiliations:** 1 Department of Biochemistry and Biophysics, “Carol Davila” University of Medicine and Pharmacy, 020021 Bucharest, Romania; alexandru.filippi@umfcd.ro; 2 Department of Specific Disciplines, “Carol Davila” University of Medicine and Pharmacy, 020021 Bucharest, Romania; justin.aurelian@umfcd.ro; 3 Department of Urology, “Prof. Dr. Th. Burghele” Clinical Hospital, 050653 Bucharest, Romania

**Keywords:** prostate cancer, Gleason score, bioinformatics, gene networks, gene signature

## Abstract

Prostate cancer (PCa) is the most prevalent non-cutaneous cancer in men. Early PCa detection has been made possible by the adoption of screening methods based on the serum prostate-specific antigen and Gleason score (GS). The aim of this study was to correlate gene expression with the differentiation level of prostate adenocarcinomas, as indicated by GS. We used data from The Cancer Genome Atlas (TCGA) and included 497 prostate cancer patients, 52 of which also had normal tissue sample sequencing data. Gene ontology analysis revealed that higher GSs were associated with greater responses to DNA damage, telomere lengthening, and cell division. Positive correlation was found with transcription factor activator of the adenovirus gene *E2* (E2F) and avian myelocytomatosis viral homolog (MYC) targets, G2M checkpoints, DNA repair, and mitotic spindles. Immune cell deconvolution revealed high M0 macrophage counts and an increase in M2 macrophages dependent on the GS. The molecular pathways most correlated with GSs were cell cycle, RNA transport, and calcium signaling (depleted). A combinatorial approach identified a set of eight genes able to differentiate by k-Nearest Neighbors (kNN) between normal tissues, low-Gleason tissues, and high-Gleason tissues with high accuracy. In conclusion, our study could be a step forward to better understanding the link between gene expression and PCa progression and aggressiveness.

## 1. Introduction

Prostate cancer is one of the most commonly diagnosed cancers in men. Worldwide, it is the second most common male malignancy, after lung cancer. Early detection opens the door to curative surgery. However, recurrence is possible in up to 30% of men who have undergone radical prostatectomy; this is frequently due to micrometastatic disease that was present at the time of surgery [1]. The prognosis of prostate cancer depends on many factors, such as age, prostate-specific antigen (PSA), pathological stage, GS, and the presence of positive margins [2]. The challenge is to identify those patients at risk for relapse and to better understand the molecular abnormalities that define tumors at risk for relapse [3].

Donald Gleason and the Veterans Administration Cooperative Urologic Research Group developed the current PCa grading system between 1966 and 1974. GS is the most commonly used staging system for prostate cancer, and it is based on a glandular differentiation scale evaluated at a microscopic level [4]. Because prostate cancer is a heterogeneous disease, GS evaluates the two most common types of growth present in the tumor. Both types are graded between 1 and 5, with 1 representing the most distinctly differentiated and 5 representing the least distinctly differentiated. The sum of the two numbers ranges between 2 and 10. When there is only one “pattern”, the GS is obtained by doubling the degree of differentiation. The reproducibility of the GS, as reported in numerous studies, was more than 70% [5]. At present, the application of Gleason grading differs significantly from the original system. Scores 2–5 are no longer assigned, and certain patterns that Gleason defined as a 6 are now graded as 7, resulting in contemporary GS 6 cancers having a better prognosis than historic score 6 cancers [6].

To address the confusion inherent in the GS system, a new grading system based on data from Johns Hopkins Hospital was proposed in 2013. This system of grades is divided into five groups, each representing a revised GS: grade group 1 (GS ≤ 6), grade group 2 (GS 3 + 4 = 7), grade group 3 (GS 4 + 3 = 7), grade group 4 (GS 8), and grade group 5 (GS 9–10) [7]. The GS, which is determined based on the surgical specimen after radical prostatectomy, is a significant prognostic factor and is likely the best indicator of the progression of the disease [3,8].

Several clinical features of prostate cancer, such as tumor cell differentiation or GS and serum PSA, are used in routine clinical practice to stratify men into low-, intermediate-, and high-risk groups for tumor recurrence after local therapy. However, the majority of patients who now undergo radical prostatectomy have low- to intermediate-risk clinical features, making determining the prognosis for these patients difficult. Gene mutations have been shown to be important in the progression of prostate cancer. Recurrent somatic mutations, copy number changes, and oncogenic structural DNA rearrangements (chromosomal abnormalities) have been identified in primary prostate cancer, metastatic prostate cancer, and metastatic, castration-resistant prostate cancer (mCRPC) [9,10,11,12].

Various online databases provide gene expression data and correlated variable clinical courses of multiple cancer types to help researchers investigate new mechanisms or treatment targets. The Cancer Genome Atlas is a project funded by the National Institutes of Health (NIH) and the National Human Genome Research Institute (NHGRI) aiming to characterize the genetic modifications occurring in cancer. The TCGA includes gene expression data from thousands of primary tumors and corresponding normal tissues from patients suffering from 33 different cancer types [9,13]. Another valuable resource in the study of human cancers is the Human Protein Atlas (HPA), a repository of immunohistochemistry (IHC) data [14,15]. With protein expression data for 15,303 genes from 40 different normal tissues and 17 types of cancer, the HPA is a comprehensive protein expression database useful for the identification of potential biomarkers for disease diagnosis and prognosis, understanding the molecular mechanisms underlying disease, and identifying new targets for therapeutic intervention. 

The variate survival time after PCa diagnosis [16] and high heterogeneity of the disease [17,18] have challenged a large number of research groups to work on this pathology and to recommend several biomarkers with the aim of improving the diagnostic, prognostic, disease progression, or clinical benefits for patients. Recent studies using available databases such as the TCGA or Gene Expression Omnibus (GEO) have focused on gene expression signature correlated with GS to predict PCa prognosis [19,20]. Mohammad and co-workers proposed a group of five modified hub genes in PCa, and two of them, signaltransduction activator of transcription 6 (*STAT6*) and sex determining region Y-Box2 (*SOX2*), appeared to be valuable biomarkers for the disease [20]. Meng and co-workers, in a study of five cohorts of patients using data from Memorial Sloan Kettering Cancer Center (MSKCC), GEO datasets [GSE116918, GSE70769, GSE70768], and the TCGA Prostate Adenocarcinoma (TCGA-PRAD) dataset, recommended eleven genes most appropriate to predict recurrence-free survival in PCa. Their bioinformatics data combined with cell culture analysis focused on two genes, steroid 5 alpha-reductase 2 (*SRD5A2*) and integrin subunit alpha 11 (*ITGA11*), as markers for cell migration and proliferation [21]. Besides the communicated gene signatures associated with PCa progression, Niu and co-workers dedicated an entire study to identifying biomarkers related to the tumor microenvironment and immune cells [22]. After analyzing samples from patients with primary PCa, xenograft mouse models, transgenic adenocarcinoma mouse prostate (TRAMP) mouse models, and data from the TCGA and MSKCC databases, Gu and co-workers showed that downregulation of the IQGAP1 motif belonging to small G proteins was strongly connected to castration-resistant and advanced PCa. In addition, the authors suggest that the Sig27gene is a novel gene signature associated with prediction of PCa recurrence [23]. Regardless of already-proposed gene signatures or diagnosis kits [24,25,26,27], there is a lack of well-validated models to help with medical decisions with respect to conservative or radical treatment in the early stages of the disease, such as in case of GS 7 (3 + 4 = 7 or 4 + 3 = 7) or oligometastasis.

In this study, using publicly available transcriptome expression data from the TCGA, we evaluated the modifications in the hub proteins, such as polo like kinase 1 (PLK1), cell division cycle 20 (CDC20), caveolin 1 (CAV1), camodulin 1 (CALM1), and p21 activated kinase 3 (PAK3), in relation to their clinical significance and determined which categories of immune cells have been associated with poorly differentiated prostate adenocarcinomas, as indicated by high Gleason scores. We also proposed an eight-gene signature, validated by immunohistochemistry staining in the HPA database, which could be of clinical interest for PCa pathology.

## 2. Results

### 2.1. Demographics and Staging

Patients in higher Gleason grade groups tended to be older, with patients in Gleason grade group 5 being an average of 4 years older than patients in Gleason grade group 1 (Table 1). Unsurprisingly, Gleason grade groups positively correlated with pathologic main tumor (T) stage and pathologic node (N) stage. Only 27.3% of patients in Gleason grade group 1 had a T stage of T3a or higher, but this percent increased steadily with the Gleason grade group, with patients in Gleason grade group 5 reaching a T3a or higher T stage in 89.3% of cases (*p* < 0.0001, chi-squared test). Similarly, regarding the N stage, no patients in Gleason grade group 1 and less than 5% patients in Gleason grade group 2 had lymph nodes involvement. The percentage of patients with lymph nodes involvement doubled for each successive Gleason grade group, reaching almost 40% in Gleason grade group 5 patients (*p* < 0.0001, chi-squared test). Of all patients, only three presented with distant metastasis, and all three had high GS values (two patients in Gleason grade group 5 and one in Gleason grade group 4).

### 2.2. Lower mRNA Levels Were Observed for a Higher Proportion of Genes in Prostate Tumor Samples Compared to Normal Tissues

**Figure 1:**
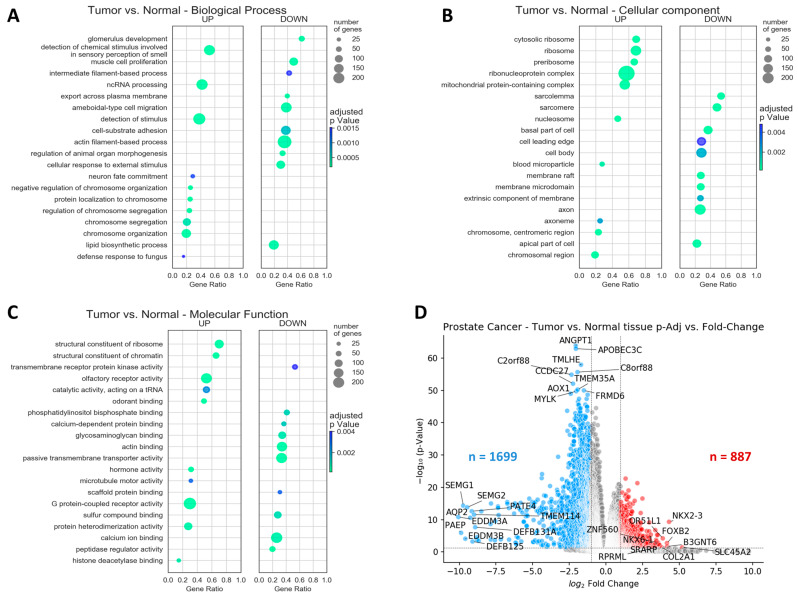
Analysis of normal and tumor genes in prostate samples. Gene ontology analysis showing the most enriched (up) or depleted (down) biological processes (**A**), cellular components (**B**), and molecular functions (**C**) in PCa compared to normal tissues. Circle sizes reflect the number of genes enriched, the x axis shows the ratio of enriched genes to all genes in the gene set, and the color denotes the false discovery rate (FDR)-adjusted *p* value. (**D**) Volcano plots of differentially expressed genes in PCa, showing log (Fold Change) plotted on the statistical significance log (FDR-adjusted *p* value). Significantly overexpressed genes are colored in red, significantly under-expressed genes are colored in blue, and labels are provided for the 10 most significantly modified (lowest *p* values), most under-expressed, and most overexpressed genes, respectively. Larger versions of Figure 1A–C are shown in Supplementary Materials (Figure S1).

Gene ontology was performed for all tumors vs. normal tissues, based on the differential expression file (Figure 1A–C, Supplementary Table S1). Tumor samples were enriched significantly with genes involved in non-coding RNA processing, ribosome components expression levels, chromosome organization and G protein-coupled activity, while depletion of the genes was correlated with sarcomere levels, cell polarity, actin filament-based processes and calcium ion binding. Gene ontology results detailed by biological processes, cellular components, and molecular functions are shown in Figure 1A–C.

Although protein production was among the most increased biological processes, and molecular functions and ribosomes were the most increased cellular components, mRNA levels were overall lower in tumor samples compared to normal tissues, with 1699 genes significantly reduced by a factor greater than two and only 887 genes significantly overexpressed, above the Fold Change = 2 threshold (Figure 1D). 

### 2.3. High GS Is Associated with Increased Proliferation and Loss of Cellular Polarization but Also Decreased Vasculature Development

**Figure 2:**
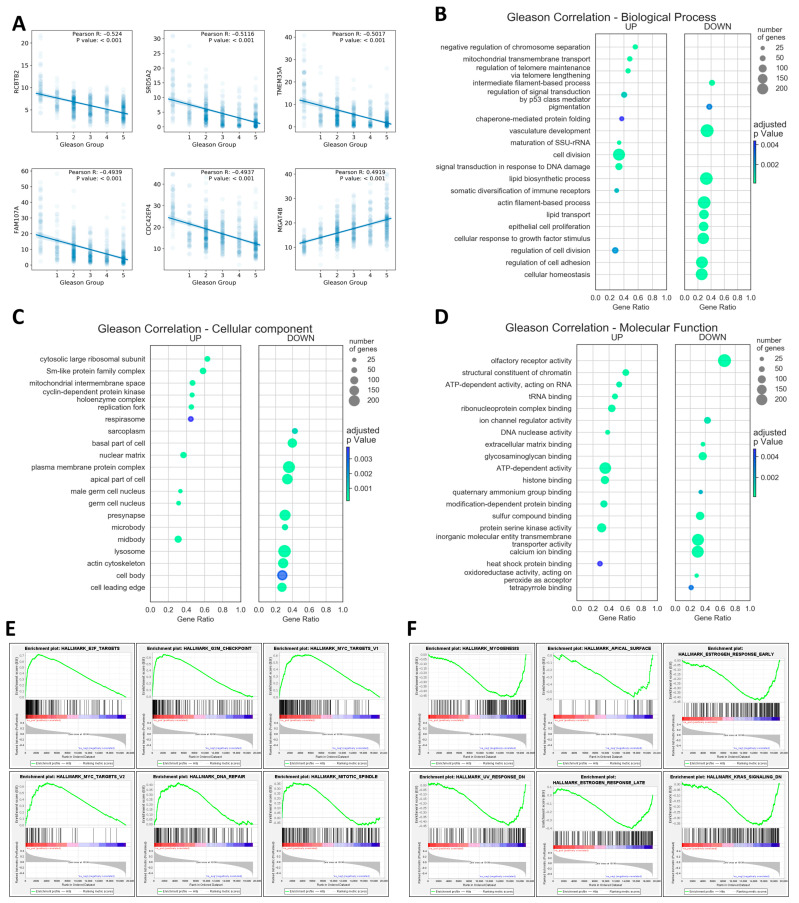
Correlation between Gleason grade groups and gene expression. Top six genes most correlated with GS (**A**). Gene ontology analysis showing the most enriched biological processes (**B**), cellular components (**C**), and molecular functions (**D**) in Gleason-correlated genes. Here, enriched or up means positively correlated with Gleason grade, and depleted or down means negatively correlated with Gleason grade. Circle sizes reflect the number of genes enriched, the x axis shows the ratio of enriched genes to all genes in the gene set, and the color denotes the FDR-adjusted *p* value. (**E**) Enriched gene sets and (**F**) depleted gene sets in higher GS tumors. Analysis was performed on the “Hallmark” collection of gene sets in gene set enrichment analysis (GSEA). Larger versions of Figure 2B–D are shown in Supplementary Materials (Figure S1).

Next, to identify processes that are enriched or depleted specifically in high GS tumors but less so in lower GS tumors, gene ontology analysis was performed using the rank of the genes, sorted from the most correlated to the least correlated with Gleason grade groups (see Figure 2A for the top six correlated genes given as example). Supplementary Table S2 shows the complete list of genes, their corresponding Pearson R value, and Benjamini–Hochberg-adjusted *p* values. This analysis found that higher Gleason grade groups correlated with increased cell division, telomeres lengthening, and response to DNA damage (regulation of signal transduction by p53 class mediator and signal transduction in response to DNA damage) and with decreased vasculature development, epithelial cell proliferation, cell adhesion, and cell polarization (Figure 2B–D).

An alternative analysis of the enriched gene sets was performed using gene set enrichment analysis, which showed that higher Gleason grade groups positively correlated with E2F and MYC targets, G2M checkpoints, DNA repair, and mitotic spindles (Figure 2E). On the other hand, higher Gleason grade groups negatively correlated with myogenesis, apical surface, estrogen response early and late, UV response down, Kirsten rat sarcoma viral homolog (KRAS) signaling down, and apical junction (Figure 2F).

### 2.4. High GS Is Associated with High Counts of M2 Macrophages

**Figure 3:**
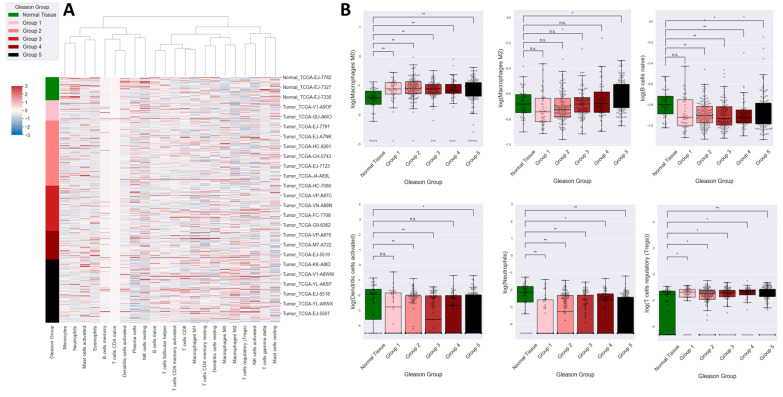
Gene analysis and immune cells in Gleason grade groups. (**A**) Heat map showing the results of immune cell populations’ enrichment in tumors from different Gleason grade groups determined with CIBERSORTx. Relative enrichment results were z-score normalized, columns were grouped by hierarchical clustering based on the ward distance, and rows show individual samples sorted by Gleason grade groups (see color legend). (**B**) Box plots of the relative immune cell enrichment for the significantly enriched cell types between tumors with different Gleason grade groups and normal tissues. Statistical significance for the comparison with normal tissues is indicated as n.s. (not significant); * *p* < 0.05, ** *p* < 0.01.

While gene ontology analysis did not find inflammation to be particularly modified in prostate cancer, nor significantly associated with GS, some differences in the immune cells makeup of tumors compared to normal tissues and between tumors of varying differentiation were observed (Figure 3). Thus, tumors from all Gleason grade groups had significantly increased numbers of regulatory T cells and lower numbers of activated dendritic cells, naïve B cells, and neutrophils compared with normal tissues. Also, relatively high numbers of M0 macrophages were found in all Gleason grade groups; however, an increase in M2 macrophages appeared to be Gleason-grade-dependent, with significantly higher levels of anti-inflammatory M2 macrophages in Gleason grade group 5 compared to normal tissues (*p* = 0.0439 after Tukey’s honest significance test).

### 2.5. Protein Networks and Pathways Involved in Gleason Progression

**Figure 4:**
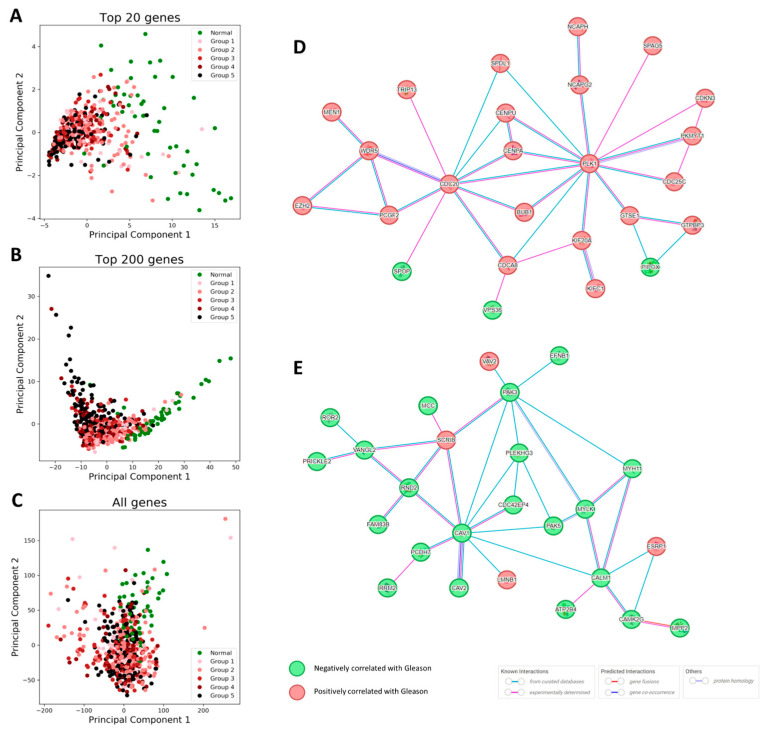
Significant separation of the genes between normal and Gleason grade groups. Principal component analysis performed on the top 20 genes correlated with the Gleason grade (**A**), top 200 genes correlated with the Gleason grade (**B**), and all genes (**C**) show the best separation in the case of the 200-gene set, with a clear transition from normal tissues to low grade groups and to high grade groups. STRING network analysis performed on the top 200 genes that correlated with the Gleason grade. There were identified two distinct networks of 25 molecules each, one positively correlated with Gleason grade, centered around CDC20 and PLK1 key nodes proteins (**D**), the other negatively correlated with Gleason grade, centered around CAV1, CALM1, and PAK3 proteins (**E**).

Principal component analysis, a method to reduce dimensionality and visualize the overlap/separation of populations, showed the best separation and a clear trajectory from normal tissues to low Gleason grade groups and high Gleason grade groups based on the 200 genes most correlated with GS. By comparison, using only the first 20 most correlated genes gave spread out populations for normal tissues and low GS, while using all genes added noise, worsening the separation of the populations (see Figure 4A–C).

Thus, the 200 genes most correlated with Gleason grade groups were used in a STRING analysis, which identified among these two clusters of interrelated molecules. The first cluster consisted of molecules mostly positively correlated with Gleason grade groups and was centered on the highly interconnected serine/threonine-protein kinase PLK1 and CDC20, having 13 and 10 associated nodes, respectively (Figure 4D). This cluster was highly enriched in proteins involved in the mitotic cell cycle (18 out of 25 proteins, *p* = 9.87 × 10^−18^) and regulation of cell cycle (17 out of 25 proteins, *p* = 1.40 × 10^−12^).

The second cluster was made up mostly of proteins negatively correlated with Gleason grade groups. Here, the most connected nodes were represented by CAV1 with 10 connections, followed by PAK3 with 7 interactions and CALM1 with 6 connections (Figure 4E). Proteins in this cluster played roles in signaling by Rho GTPases (14/25, *p* = 2.38 × 10^−11^), signal transduction (18/25 proteins *p* = 1.13 × 10^−8^), focal adhesion (6/25, *p* = 5.26 × 10^−5^), and regulation of the actin cytoskeleton (5/25, *p* = 0.0011).

**Figure 5:**
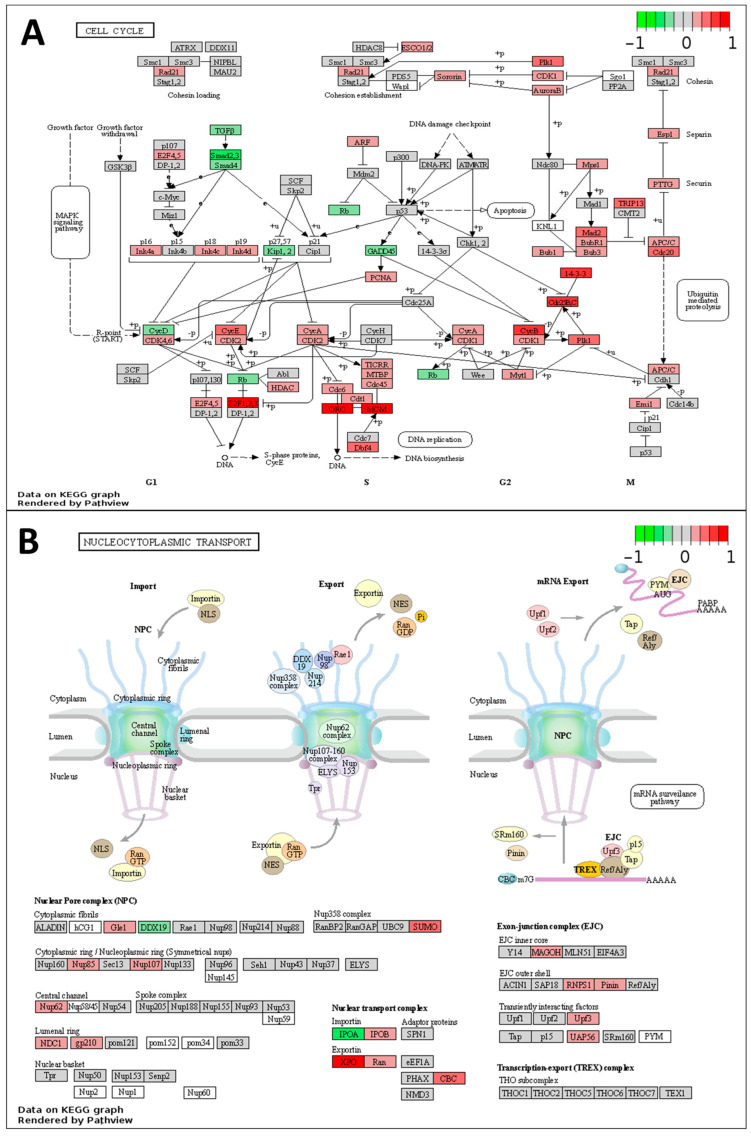
Signaling pathways upregulated in higher Gleason grade groups. Signaling pathways hsa04110 “Cell cycle” (**A**) hsa03013 RNA transport (**B**). Node color shows the Pearson R coefficient for the correlation between protein expression level and Gleason grade groups, with red signifying positive correlation with Gleason grade groups and green signifying negative correlation.

**Figure 6:**
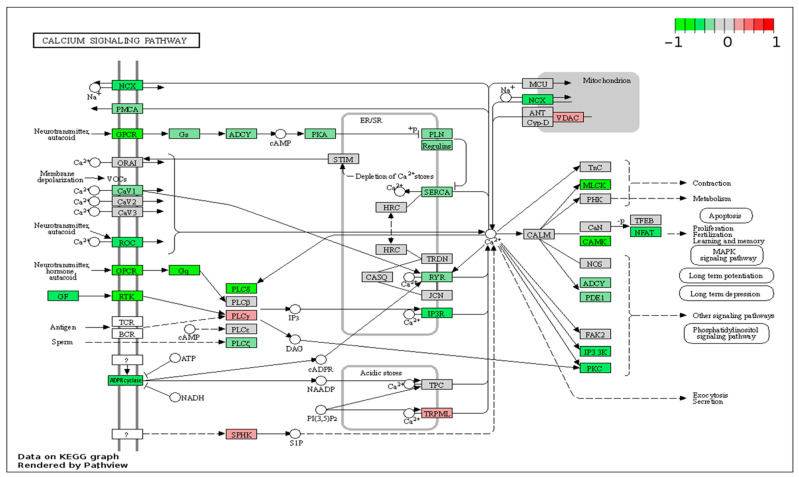
Calcium signaling pathway (hsa04020) is enriched in proteins negatively correlated with GS. Node color shows the Pearson R coefficient between expression level and Gleason grade groups, with red signifying positive correlation with Gleason grade groups and green signifying negative correlation.

The molecular pathways most correlated with the Gleason grade groups as identified by the Pathview package in R/Bioconductor were cell cycle (enriched, *p* = 0.0016), RNA transport (enriched, *p* = 0.0017), and calcium signaling (depleted, *p* = 0.0046) pathways. Correlations with Gleason grade groups are plotted for the individual proteins overexpressed in the cell cycle pathway and RNA transport (Figure 5A,B). and For calcium signaling (Figure 6) most individual proteins were under-expressed in higher compared to lower grade Gleason tumors.

### 2.6. Selected Gene Signature for Gleason Grade Groups

**Figure 7:**
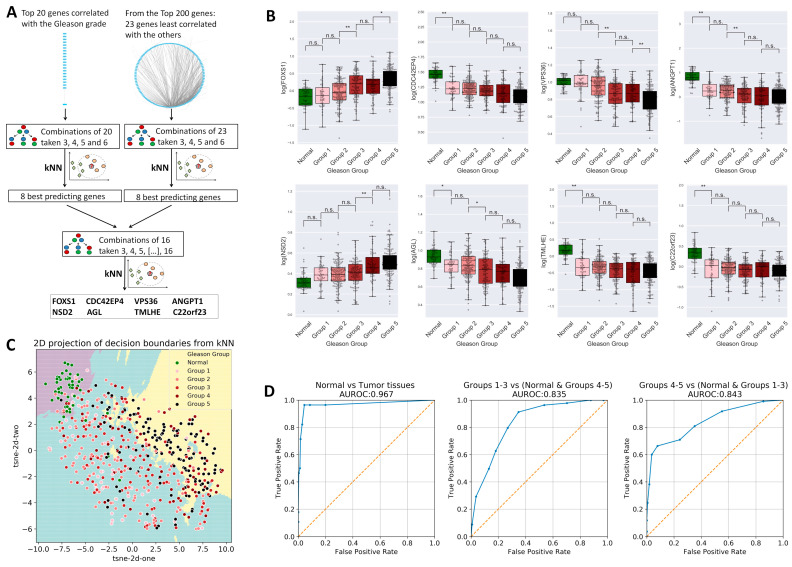
Gene selection for Gleason grade group prediction. (**A**) Strategy for gene selection: top 20 genes correlated with Gleason grade were used in successive combinations of 3, 4, 5, or 6 to identify the genes most predictive of Gleason grade groups in kNN analyses. Separately, a network analysis revealed the 23 least inter-correlated genes from the top 200 genes correlated with Gleason grade, and these were used in successive combinations of 3, 4, 5, or 6 to identify the genes most predictive of Gleason grade groups in kNN analyses, as described above. The results were sorted for accuracy, and the best 8 predicting genes from each set of candidate genes were pooled. A new combination followed by kNN was performed, and the best predictor genes were selected as gene signature for Gleason grade. (**B**) Analysis of variance (ANOVA) of the selected genes, showing differences between increasing Gleason grade groups (*p < 0.05, **p < 0.01). (**C**) t-SNE 2d projection of the eight dimensional space of the selected parameters Decision boundaries by kNN (*n* = 10) are shown. (**D**) Receiver operating characteristic curves (ROC) showing the performance of the classification method for the identification of normal tissues, low-Gleason (grade groups 1–3), and high-Gleason (grade groups 4–5).

In order to identify a gene signature predictive of Gleason grade, samples were grouped in normal tissues, low-Gleason (Gleason grade groups 1–3), and high-Gleason (Gleason grade groups 4–5), and then the top 20 genes correlated with Gleason grade were queried in kNN analyses, in successive combinations (see Figure 7A). Because the top 20 genes were highly correlated among them and thus each gene carried similar information to the others in the set, another selection approach used 23 genes selected from the top 200 genes based on their low inter-correlations as determined in a network analysis performed in Cytoscape 3.7. 

**Figure 8:**
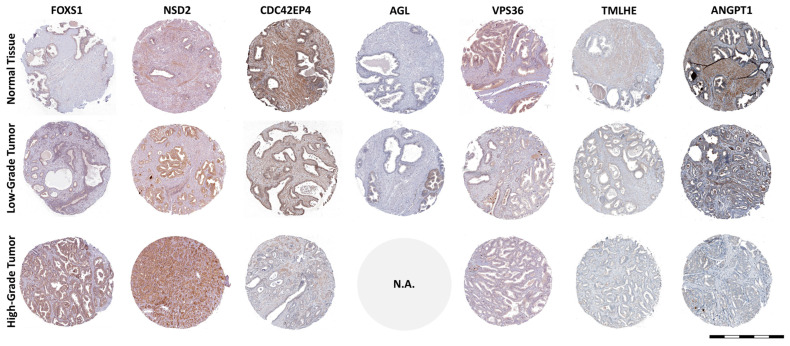
Validation of *FOXS1*, *NSD2*, *CDC42EP4*, *AGL*, *VPS36*, *TMLHE*, and *ANGPT1* genes by immunohistochemistry staining in normal prostate and PCa samples using the HPA database. Data not available (N.A.) in the case of the *AGL* gene for high-grade tumors and *C22orf23* gene Scale bar in the lower right corner represents 1 mm.

From each of the two gene sets, the first eight genes, which consistently yielded high global accuracy scores in combinations with other genes, were selected and pooled. The resulting 16-gene set was further tested using the same combinatorial approach; finally, genes forkhead box protein S1 (*FOXS1*), nuclear receptor binding SET domain protein 2 (*NSD2*), cell division cycle 42 effector protein 4 (*CDC42EP4*), amylo-alpha-1, 6-glucosidase, 4-alpha glucanotransferase (*AGL*), vacuolar protein sorting 36 homolog (*VPS36*)*,* trimethyllysine hydroxylase epsilon (*TMLHE*), angiopoietin 1 (*ANGPT1*), and chromosome 22 open reading frame 23 (*C22orf23*) were selected as best predicting Gleason grade groups. Based on these genes, normal tissues could be identified with an area under the receiver operating characteristic curve (AUROC) of 0.967 and the samples with low-Gleason and high-Gleason grades were identified with AUROC values of about 0.84 each (Figure 7D). The separation between samples with different Gleason grade groups based on the selected eight signature genes can also be observed in Figure 7C, showing the t-distributed Stochastic Neighbor Embedding (t-SNE) 2D projection of the decision space. Increased expression of *FOXS1* and *NSD2* genes significantly correlated with high-grade GS (Figure 7) was validated in the HPA databases; the same is true in cases of inverse correlation between the other six genes and GS (Figure 8). 

## 3. Discussion

In this study, we investigated the correlation between gene expression profiles and GS in samples from prostate cancer patients using transcriptomic data. We evaluated 497 prostate cancer patients, 52 of which had normal tissue sample sequencing data recorded. We identified 247 patients with GS 7 (group 2 and group 3), 64 patients with GS 8 (group 4), and 141 patients in group 5, meaning patients with GS 9 and 10. In group 5, more than 89% of the cases had a T3 or T4 stage. Next, we performed gene ontology analysis, which showed that higher Gleason grade groups correlate with increased cell division, telomeres lengthening, and response to DNA damage. Enriched gene set analysis demonstrated positive correlation between higher Gleason grade groups and DNA repair processes, whereas negative correlation was found in relation to myogenesis and estrogen responses. Regardless of GS, prostate tumors have on average a higher number of M0 macrophages and regulatory T cells, while neutrophils, naïve B, and dendritic cells are at lower levels. Further using STRING network analysis, we observed that *PLK1*, *CDC20*, *CAV1*, *CALM1*, and *PAK*3 genes are central nodes in the networks formed by the genes most correlated with high GS. The signaling pathways most consistently correlated with high GS are cell cycle, RNA transport (increased), and calcium signaling (decreased) pathways. A novel gene expression signature predictive of Gleason grade included eight genes, namely *FOXS1*, *NSD2*, *CDC42EP4*, *AGL*, *VPS36*, *TMLHE*, *ANGPT1*, and *C22orf23*. Additionally, validation of the gene expression signature was carried out by immunohistochemistry staining in the HPA database. 

Our data pointed out significantly lower total levels of mRNA in PCa samples compared to the normal ones (Figure 1D). On a further literature study of the topic, B. Ozer and U. Sezerman also remarked that “lowest significance and fold changes were observed at the prostate cancer dataset” compared to colon, breast, and thyroid cancers [28]. In addition, another recent study by W. Niu, T. Zhang, and L. Ma also identified fewer upregulated genes compared to downregulated genes in prostate cancer [22]. The most significantly reduced levels of gene expression were noticed for *ANGPT1*, a gene coding for angiopoietin 1, and *APOBEC3C*, a gene coding for an isoform of apolipoprotein B. These results are in agreement with low levels of RNA for the lipid biosynthetic process (Figure 1A) and reduced vasculature development (Figure 2B). Higher levels of mRNA are associated with poor prognosis in several types of cancer [29]; nevertheless, lower expression of mRNA in the case of tumor protein P53 (*TP53*) and retinoblastoma 1 (*RB1*), two tumor suppressor genes, can facilitate resistance to ADT in PCa [30]. In addition, a combination of *TP53* and *RB1* loss was reported in a subtype of PCa that lacks AR pathway activity [31], data which are in line with low expression of genes involved in response to androgen in advanced PCa [32]. 

As whole transcriptome sequencing becomes more and more affordable and data become freely available, transcriptome-wide studies not restricted to preselected genes can be easily performed [33]. Thus, in this study, all protein coding genes were ranked for their correlation with Gleason grade groups (Figure 2). Five the most downregulated genes were regulator of chromosome condensation and BTB domain 2 (*RCBTB2*), steroid 5 alpha-reductase 2 (*SRD5A2*), transmembrane protein 35A/ nicotinic acetylcholine receptor regulator (*TMEM35A*), family with sequence similarity 107 member A/ actin-associated protein (*FAM107A*), and Cdc42 effector protein 4/binder of RhoGTPases (*CDC42EP4*), while the most upregulated was alpha-1, 3-monnosyl-glycoprotein 4-beta-N-acetylglucosaminyl transferase (*MGAT4B*). The full list of protein coding genes sorted by their correlation with Gleason grade groups is presented in Supplementary Table S2. Ross-Adams and his co-workers demonstrated that the *RCBTB2* gene, in addition to the other five genes, was associated with prostate cancer [34]. At the same time, *MGAT4B*, a gene responsible for glycosyltransferase synthesis, which is involved in aberrant glycosylation in cancer [35], appears to be associated to prostate cancer risk [36]. 

Treatment for prostate cancers is based on their dependence on androgen for growth, survival, and development. Prostate cancer cell death or cell cycle arrest is brought on by androgen ablation. Androgen receptors (ARs) are members of the nuclear receptor superfamily and act as ligand-dependent transcription factors [37]. It is evident that cell context affects ARs’ ability to subsequently trigger a gene expression program that encourages cell cycle progression. Since PCa is considered an androgen-dependent tumor, the role of estrogen activity was to some extent neglected. Earlier studies proposed estrogens or anti-estrogen agents as hormonal therapy in androgen-dependent prostate cancer [38,39]. Nevertheless, several lines of evidence showed the association between estrogens and prostate carcinogenesis through a number of mechanisms such as receptor mediation [40], genotoxic effects of estrogen metabolites [41], bypassing the AR signaling pathways [42], or androgen aromatization to estrogens [43]. In a normal prostate, both estrogen receptor alpha (ER-α) and ER-β are expressed, while ER-β expression is decreased in malignancy, indicating that ER-β might have a tumor suppressor function [44]. Our data demonstrated a low level of estrogen gene expression in PCa (Figure 2), which might be in line with reduced ER-β activity. In prostate cancer cell lines, the anti-proliferative activity of ER-β has been shown by the downregulation of key oncoproteins including phosphoinositide 3-kinase (PI3K), c-Myc, and Cyclin E and the upregulation of tumor suppressor genes such as phosphatase and tensin homolog (*PTEN*) and forhead box O3 (*FOXO3*), supporting the idea that ER-β agonists might be considered for inhibiting the malignant processes [44].

In the progression of cancer pathology, the blood vessels are common routes for tumor cells to spread from the primary site, while their excessive growth nourishes the distant tumors [45]. The therapeutic approach includes first targeting the receptors of vascular endothelial growth factor (VEGFR) with immunotherapy (bevacizumab) or administration of protein kinase inhibitors (sunitinib) approved for advanced stages of cancer [46]. Nevertheless, clinical trials with inhibitors of VEGFR did not prolong disease-free survival, and the tumors demonstrated several mechanisms to escape the therapeutic approaches [47]. The same is true in the case of PCa, where the angiogenesis inhibition did not increase overall survival [48]. Our data demonstrated downregulation of the genes involved in the vasculature development associated with high GS (Figure 2B). This observation could suggest other possible explanations for the low response to anti-angiogenetic therapy. 

A characteristic of the tumor microenvironment is hypoxia, a feature associated with the inflammatory process and angiogenesis [49]. Among immune cells, tumor-associated macrophages (TAM) prefer to hide in hypoxic media, and their abundance is associated with poor clinical outcome [50]. In prostate cancer, macrophages with the M2 phenotype are involved in metastasis, proliferation, immune suppression, and therapeutic resistance [51,52]. Our data demonstrated the association between macrophages with the M2 phenotype and a high Gleason score (Figure 3). A recent idea suggests that TAM might represent a therapeutic target in cancer [53], and possible mechanisms consist of depletion of total TAM, reprograming M2-like phenotypes to M1-like phenotypes, or inhibition of crosstalk between TAM and tumor cells [54].

Our data indicated that *PLK1*, *CDC20*, *CAV1*, *CALM1*, and *PAK3* genes are central nodes in the networks formed by the genes most correlated with a high GS, with *CDC20* and *PLK1* being overexpressed, while *CAV1*, *CALM1*, and *PAK3* are under-expressed (Figure 4). In normal cells, the CDC20 protein is responsible for complex activities during mitosis [55], and it was shown to be overexpressed in various types of human cancer [56]. In prostate cancer, CDC20 has been reported to function as an independent predictor for biochemical recurrence [57]. Wu et al. shown recently that depletion of CDC20 significantly enhances anti-tumor immunity by promoting the infiltration of CD8+ T lymphocytes dependent on the existence of gasdermin family member E, an apoptosis mediator. In addition, the authors indicated that synergic activity was noticed between immune checkpoints inhibition and Apcin, a small molecular inhibitor of CDC20 [58]. PLK1 regulates many aspects of the cell cycle, and its dysregulation is a common feature of cancer. PLK1 is overexpressed in prostate cancer and has been linked to higher tumor grades, implying that PLK1 plays a role in PCa tumorigenesis and progression [59].

A detailed network of the calcium signaling pathway obtained with the Pathview package represents a novel observation in PCa studies (Figure 6). It was previously shown that expression of calcium/calmodulin-dependent kinase II (*CaMK2*) genes is under AR control, with active AR in the presence of androgens inhibiting *CaMK2* gene expression and low AR activity resulting in enhanced expression of *CaMK2* [60]. AR expression depends on the Gleason pattern, with maximum levels in Gleason pattern 4 [61]. In our results, both calmodulin and *CAMK2* genes were negatively correlated with GS, being among the top 200 correlated genes (Figure 4E). Interestingly, in PCa cells from bone metastases, but not in primary PCa, CAMK2 regulates the production of the active, cleaved form of Notch1, which in turn controls the *MYC* oncogene [62]. Another family of calcium-dependent molecules, the nuclear factor of activated T cells (NFAT) family of transcription factors, has members with roles in cell cycle progression, cell differentiation, and apoptosis, acting as either oncogenes or tumor suppressor genes in different types of cells [63]. In our results, the more studied NFAT1 and NFAT2 were not associated with GS, while NFAT3, NFAT4, and NFAT5 were negatively correlated with the Gleason grade groups (Figure 6 and Supplementary Table S2).

Based on the genes linked to cell cycle proliferation [33], prostate tumorigenesis [26], and PCa outcomes [25,64,65], a number of prior studies have created prognostic gene expression signatures. The cell cycle progression score [33], Prolaris^®^, was shown to have predictive value for the results in radical prostatectomy specimens [66] and needle biopsy [67,68]. The prognoses for biochemical recurrence [25], metastatic progression [69], and overall survival [67,68] have all been improved. Several previous studies developed 16- or even 157-gene signatures based on gene prioritization [70,71]. Moreover, due to the benefit of large free available sequencing databases, other groups proposed a 49-gene expression signature based on correlation between GS and PCa recurrence [32], a 4-gene signature predictive for biochemical recurrence [19], or an 11-gene expression signature correlated with recurrence-free survival in 1046 patients from five databases [21]. If Figure 1D showed the most correlated genes with PCa irrespective of GS, and Figure 2A indicated the most modified genes correlated with GS without taking into account redundancies in the activity of the genes, the gene expression signature came to discriminate normal, low-Gleason, and high-Gleason prostate tissues with high precision (Figure 7). Our data suggest an eight-gene expression signature (*FOXS1*, *NSD2*, *CDC42EP4*, *AGL*, *VPS36*, *TMLHE*, *ANGPT1*, and *C22orf23*) required for cell growth and survival, glycogen degradation, distant metastasis, or angiogenesis (Table 2). Different technical approaches to analyzing data [71], diverse combinations of databases [21], different experimental design, usage of different outcome endpoints or predictors of outcome such as GS or BCR, and survival of the patients [19] may be the main reasons for obtaining a variety of gene expression signatures reported in PCa.

**Figure 9:**
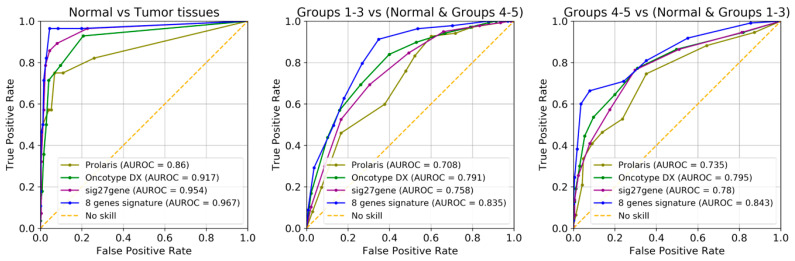
Comparison of GS predictive power for the eight-gene signature gene set described here versus three other gene sets described in the literature. Receiver operating characteristic curves (ROC) showing the performance of the classification method for the identification of normal tissues, low-Gleason tissues (grade groups 1–3), and high-Gleason tissues (grade groups 4–5). AUROC values for discriminating normal tissues, low-Gleason tissues, and high-Gleason tissues are presented for each set of genes.

As the tissue differentiation measured by GS is an important determinant of future disease evolution, we explored the possibility that gene scores previously developed to predict biochemical recurrence, such as Prolaris^®^ (31 testing genes [33]), Oncotype DX^®^ (12 testing genes [25]), and Sig27gene (27 genes [23]), could also perform well in tests to determine tissue differentiation. We used these gene sets in a kNN analysis identical to that used to determine the performance of the here-identified eight-gene signature for GS prediction (Figure 9). One can see that the eight-gene signature appears to be a better indicator in GS prediction, according to its purpose. It is remarkable how well the other three sets of genes performed in GS prognosis, although they were developed for predicting recurrence and not to assay tissue differentiation. 

The methodology used in this study has the advantage of being a comprehensive analysis of the changes associated with tumor differentiation, as indicated by Gleason grade, and of providing novel findings such as the immune cell makeup of tumors of varying differentiation and the association of specific genes with Gleason grades. Such findings have potential clinical applications in developing a novel gene expression signature for classifying prostate tissues with utility in improving diagnosis and prognosis. The validation of the observed genetic expression in the differently differentiated tissues using protein expression data from the Human Protein Atlas further adds credibility to the results and enhances their translational relevance. However, a disadvantage of this study is that its findings may not be universally applicable, as they are based on specific datasets and populations and were obtained in retrospectively. Translating these findings into clinical practice requires additional research and validation, such as a prospective study of the utility of the specific gene signature identified here in predicting the overall outcomes of prostate cancer patients. 

## 4. Materials and Methods

### 4.1. Data Used

Transcriptome profiling data for prostate cancer along with the corresponding clinical information available in the public database TCGA was downloaded from the Genomic Data Commons (GDC) portal (https://portal.gdc.cancer.gov/, accessed on 30 October 2023). In total, 497 prostate cancer patients were included in this study, 52 of which also had normal tissue sample sequencing data recorded. Using Python, all sequencing and clinical data were integrated into a single database according to the download manifest file, and patients were divided into Gleason grade groups based on the recorded GS. If not explicitly mentioned otherwise, all analyses in the following sections were performed in Python 3 using the pandas package [80] for database operations, and matplotlib [81] and seaborn [82] packages for visualizations. Data regarding immunohistochemically validation of gene expression signature for prostate cancer are from public database Human Protein Atlas (HPA, https://www.proteinatlas.org/, accessed on 29 January 2024). 

### 4.2. Differential Expression and Correlations

The fragments per kilobase of transcript per million mapped reads upper quartile (FPKM_UQ) data for protein coding genes were analyzed with unpaired *t* tests, and log2 Fold Change values were calculated to obtain the differential expression file. Additionally, an alternative analysis was made by analyzing the correlation of the FPKM_UQ values for each gene with the Gleason group by the Pearson’s correlation test. In both cases, the *p* values were adjusted for multiple comparisons using the false discovery rate Benjamini–Hochberg correction method.

### 4.3. Gene Ontology

Gene ontology analysis was performed using the clusterProfiler package in R Studio v. 2023.09.1+494 built on top of R 4.3.2. Gene sets with less than 50 members were filtered out, and the redundancy was further reduced using REVIGO (http://revigo.irb.hr/, accessed on 29 November 2023, Ruđer Bošković Institute, Zagreb, Croatia). The top 20 enriched gene sets (10 up and 10 down) for each of biological process, molecular function, and cellular component were visualized using Python 3. The differentially expressed genes were represented in matplotlib by volcano plots as log2 Fold Change (FC) vs. log2 *p* value (adjusted for multiple comparisons), using significant threshold values for both FC (0.5 and 2) and *p* values (0.05), and labels for the most significantly modified genes and for the most overexpressed or under-expressed genes were added using the adjustText library. 

An alternative analysis of the enriched gene sets was performed using the Gene Set Enrichment Analysis (GSEA v.4.10, Broad Institute, Cambridge, MA, USA) software on the Hallmark collection from Molecular Signatures Database (MSigDB https://www.gsea-msigdb.org/gsea/index.jsp, accessed on 13 November 2013).

### 4.4. Cell Type Enrichment Analysis

The relative abundance of different cell types in each sample was assessed by the cell enrichment assay algorithm from CIBERSORTx (Stanford University, Stanford, CA, USA), using the LM22 matrix. The cluster plot showing the relative cell abundance in the Gleason groups and linking cell types that tend to correlate was graphed in Python version 3.7.4 using the Seaborn package. The relative enrichment between Gleason groups was assessed by ANOVA, correcting for the number of comparisons by Tukey’s honest discovery test (statsmodels [83]).

### 4.5. Protein Networks

The 200 genes most correlated with Gleason grade were analyzed in a STRING analysis for their interactions (https://string-db.org/, accessed on 4 December 2023) [84]. Genes were linked based on experimentally determined data, data from curated databases, known gene fusion, and co-occurrences with a degree of confidence larger than 0.4 (medium). To identify individual clusters, MCL clustering analysis with an inflation parameter of 1.3 and genes in these clusters were assayed for their functions and plotted with colors indicating their correlation with the GS. 

### 4.6. Signaling Pathways

The signaling pathways most significantly altered in association with higher Gleason grade groups were assayed using the R/Bioconductor Pathview package [85] through the web-based interface available at https://pathview.uncc.edu/ (accessed on 2 November 2013), using as input data the list of all genes along with their corresponding Pearson R for the FPKM_UQ with Gleason grade group correlations.

### 4.7. Gene Selection and k-Nearest Neighbors (kNN) Analyses 

To assess the value of varying gene combinations in identifying Gleason grades, tissues were divided into 3 groups: normal tissues, Gleason grade groups 1–3, and Gleason Grade groups 4–5. Two approaches were used for the selection of genes: first, combinations of the top 20 genes correlated with Gleason grade were assessed by kNN analyses, and 8 genes with prediction value were provided. In a second approach, the top 200 genes correlated with Gleason grade were assessed for their inter-correlations, and pairs of genes with Pearson R < 0.2 were used to construct a network. The network analysis in Cytoscape 3.7 identified the 23 genes with the highest degree (number of directed genes > 10), which were employed in combinations and analyzed by kNN, yielding an additional 8 genes. kNN analysis was performed on the selected 16 genes, and the combination providing the best accuracy was chosen as the gene signature for Gleason grade. In all instances above, kNN was performed by first randomly dividing the dataset into training and validation groups (50:50), fitting the model with the training data (with 10 neighbors and Euclidean distance as hyperparameters), and then predicting the groups of the unseen validation data points using the sklearn package in Python [32]. Global accuracy was used as performance indicator. 

## 5. Conclusions

Taken together, we evaluated biological processes, molecular functions, and cellular components in 52 normal and 497 PCa RNA-sequence samples with a focus on the most under- and overexpressed genes. In addition, an analysis of five Gleason grade groups and top genes most correlated with GS was carried out. The highest-grade group displayed the strongest infiltration with M2 macrophages, while all grade groups displayed the presence of M0 macrophages. Using STRING analysis, two main networks of genes, one having a GS-positive correlation with focus on key node proteins such as CDC20 and PLK1 and the other one negatively correlated with GS, centered on CAV1, CALM, and PAK3 key node proteins, are presented. Signaling pathways extracted by the Pathview package showed increased expression of cell cycle and RNA transport pathways, and as a novelty, a complex calcium signaling pathway that decreased in PCa samples was presented. Even if, in some diseases, a higher overlapping between normal and pathological genes is seen, our data indicated clear gene separation between normal and PCa samples. Out of the top 200 genes correlated with GS, a novel eight-gene expression signature of *FOXS1, NSD2, CDC42EP4*, *AGL*, *VPS36*, *TMLHE*, *ANGPT1*, and *C22orf23* for classifying normal tissues, low-Gleason tissues, and high-Gleason tissues was extracted. Furthermore, the validation of gene expression signature was carried out using the HPA. 

A few limitations in this study need to be acknowledged. Since this study was conducted using retrospective data obtained from public datasets, further prospective results are needed to support each other. The advantage of free available databases in the case of non-communicable diseases brings a lot of hope in identifying new useful molecular targets or biomarkers; however, such a high amount of information can hinder data interpretation and clinical implementation. Future studies are required to clarify the detailed molecular mechanisms and functions of the genes in the case of difficult medical decisions regarding GS 7 (3 + 4 = 7)/ (4 + 3 = 7) and oligometastasis. Additional knowledge about genetic profile from circulating tumor DNA or circulating tumor cells using liquid biopsies as surrogate techniques may bring psychological comfort to the patients with PCa compared to more invasive procedures. In the translational field of medicine, the journey from bench to bedside is still an ongoing process, and understanding the genetic architecture of a given complex disease is essential for clarifying the pathological mechanisms, prevention of the malady, and clinical approaches.

## Figures and Tables

**Table 1:** Clinical data of the patients with PCa from TCGA included in this study. The table shows the clinical characteristics of patients with PCa divided into five grading groups according to the International Society of Urological Pathology (ISUP) [7].

	Normal Tissues	Group 1	Group 2	Group 3	Group 4	Group 5
Number	52	45	146	101	64	141
Age (mean + stdev)	60.3 ± 7.3	58.2 ± 7.6	59.8 ± 6.8	61.4 ± 6.5	61.7 ± 7	62.7 ± 6.3
Ethnicity						
Asian	0 (0%)	0 (0%)	3 (2.1%)	3 (3%)	1 (1.6%)	5 (3.5%)
African American	7 (13.5%)	11 (24.4%)	26 (17.8%)	12 (11.9%)	2 (3.1%)	6 (4.3%)
Caucasian	44 (84.6%)	30 (66.7%)	116 (79.5%)	82 (81.2%)	59 (92.2%)	126 (89.4%)
Other or not reported	1 (1.9%)	4 (8.9%)	1 (0.7%)	4 (4%)	2 (3.1%)	4 (2.8%)
Pathologic T						
T2a	-	5 (11.4%)	7 (4.9%)	1 (1%)	0 (0%)	0 (0%)
T2b	-	2 (4.5%)	4 (2.8%)	0 (0%)	3 (4.8%)	1 (0.7%)
T2c	-	25 (56.8%)	84 (58.3%)	31 (31%)	16 (25.8%)	8 (5.7%)
T3a	-	11 (25%)	40 (27.8%)	45 (45%)	23 (37.1%)	39 (27.9%)
T3b	-	1 (2.3%)	8 (5.6%)	21 (21%)	19 (30.6%)	86 (61.4%)
T4	-	0 (0%)	1 (0.7%)	2 (2%)	1 (1.6%)	6 (4.3%)
Pathologic N						
N0	-	23 (100%)	112 (95.7%)	83 (90.2%)	47 (77%)	80 (61.1%)
N1	-	0 (0%)	5 (4.3%)	9 (9.8%)	14 (23%)	51 (38.9%)
Clinical M						
M0	-	38 (100%)	136 (100%)	93 (100%)	58 (98.3%)	130 (98.5%)
M1a	-	0 (0%)	0 (0%)	0 (0%)	1 (1.7%)	0 (0%)
M1b	-	0 (0%)	0 (0%)	0 (0%)	0 (0%)	1 (0.8%)
M1c	-	0 (0%)	0 (0%)	0 (0%)	0 (0%)	1 (0.8%)

**Table 2:** Gene expression signature for classifying normal tissues, low-Gleason tissues, and high-Gleason tissues.

Gene Symbol	Gene Name	Function in PCa/Cancer	References
*FOXS1*	Forkhead Box Protein S1	Mutated in PCa; required for cell growth and survival	[72]
*NSD2*	Nuclear Receptor Binding SET Domain Protein 2	Drives metastatic progression, maintains neuroendocrine phenotype of PCa	[73,74]
*CDC42 EP4*	Cell Division Cycle 42 Effector Protein 4	Inhibits proliferation and invasion	[75]
*AGL*	Amylo-Alpha-1, 6-Glucosidase, 4-Alpha Glucanotransferase/glycogen debranching enzyme	Involved in glycogen degradation *	[76]
*VPS36*	Vacuolar Protein Sorting 36 Homolog	Predictive for reduced BCR-free survival; associated with distant metastasis	[77]
*TMLHE*	Trimethyllysine Hydroxylase, Epsilon	Methylated in PCa	[78]
*ANGPT1*	Angiopoietin 1	Involved in angiogenesis *	[79]
*C22orf23*	Chromosome 22 Open Reading Frame 23	NA	NA

Legend: *, data not available for patients with PCa; BCR, biochemical recurrence; SET, Su(var)3–9, enhancer-of-zeste and Trithorax; NA, not available.

## Data Availability

Data are contained within the article and Supplementary Materials.

## Supplementary Materials

The supporting information can be downloaded at: https://www.mdpi.com/article/10.3390/ijms25073626/s1.
