# Supplementary material for: Analysis of the Gene Networks and Pathways Correlated with Tissue Differentiation in Prostate Cancer
Source: Int J Mol Sci. 2024 Mar 24;25(7):3626. doi: 10.3390/ijms25073626 (PMC11011430; doi:10.3390/ijms25073626)
Supplement: Supplementary file 1 [file ijms-25-03626-s001.zip › ijms-2874248_SupplFigureS1.pdf]

*Article*

# Analysis of the Gene Networks and Pathways Correlated with Tissue Differentiation in Prostate Cancer

**Supplementary materials**

**Figure S1.** Gene ontology in PCa pathology

For better visualization of the labels in gene ontology analysis, larger version of Figures 1A-C, 2B-D are shown in supplementary materials.

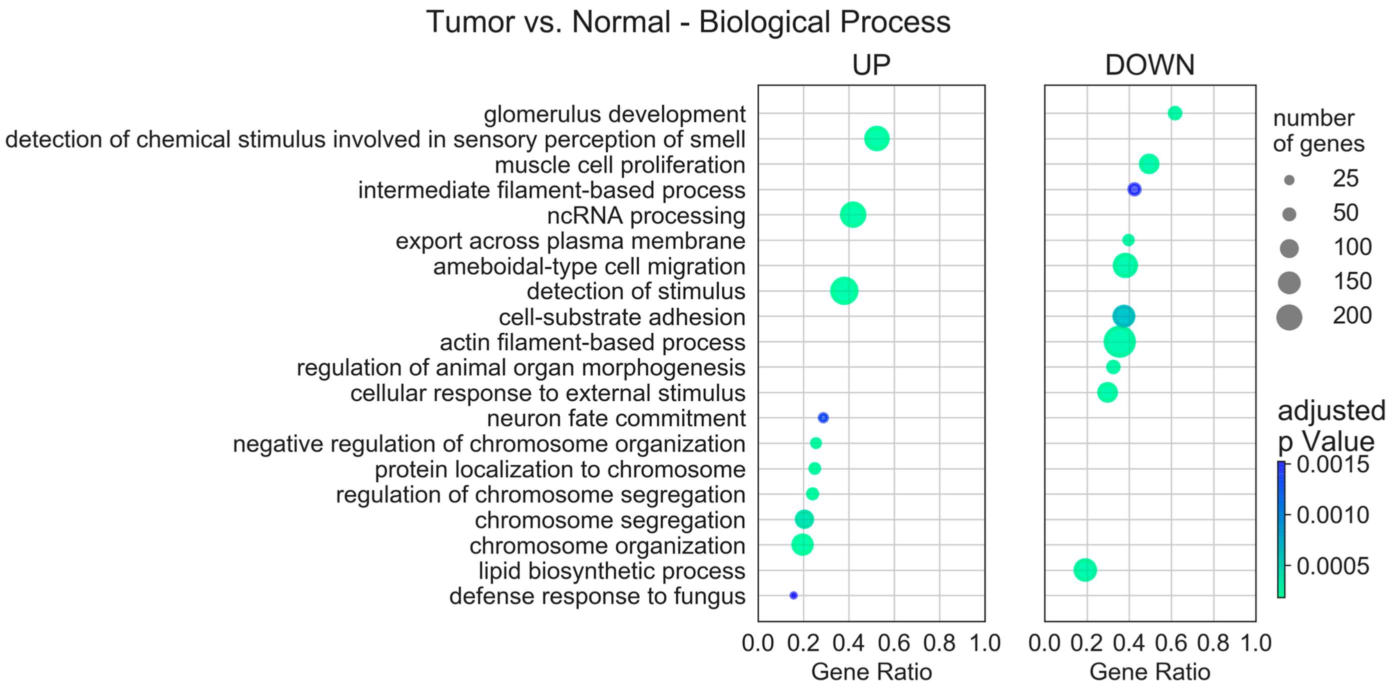


**Figure 1A.** Gene ontology analysis showing upregulated and downregulated set of genes associated with biological processes in normal and PCa samples.

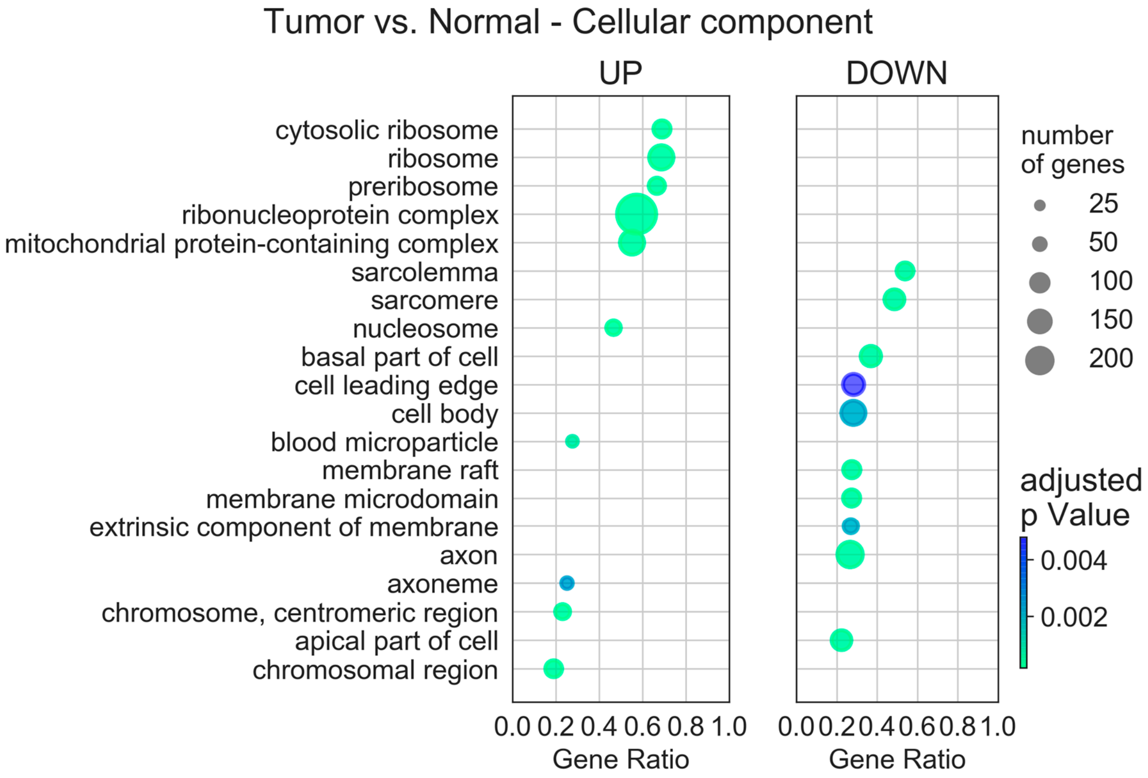


**Figure 1B.** Gene ontology analysis showing upregulated and downregulated set of genes associated with cellular components in normal and PCa samples.

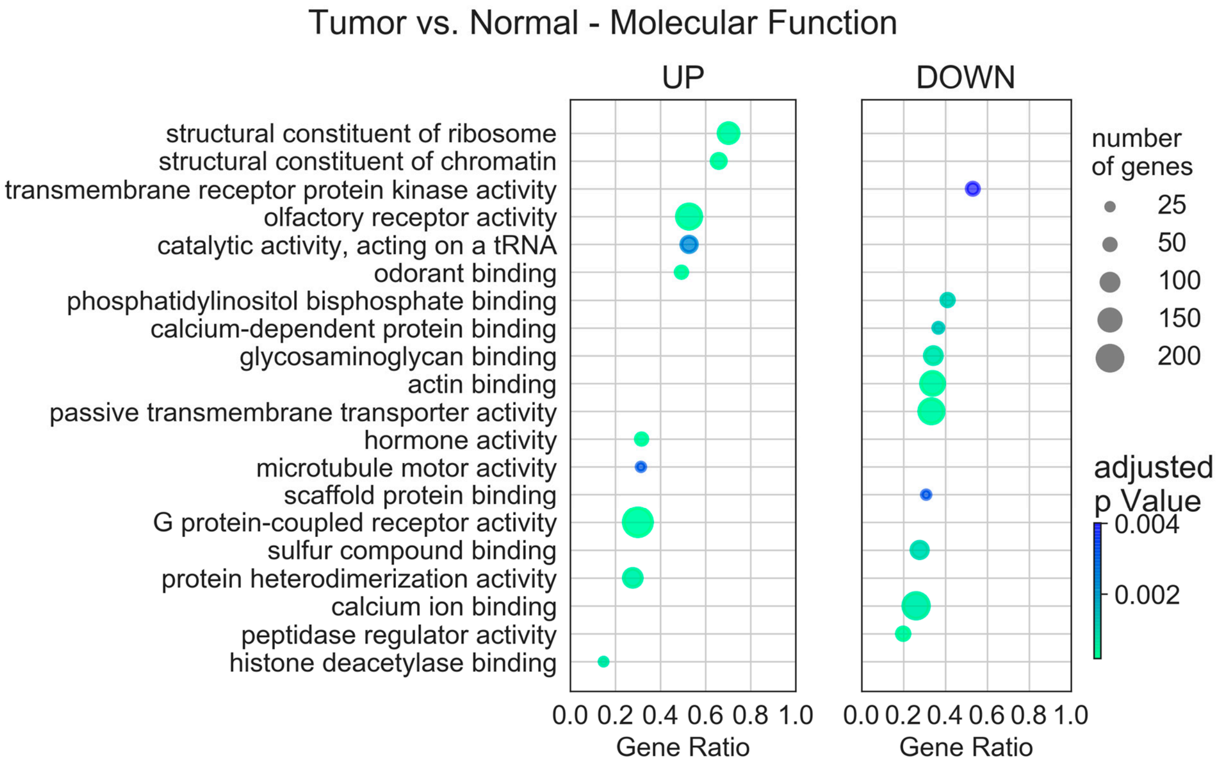


**Figure 1C.** Gene ontology analysis showing upregulated and downregulated set of genes associated with molecular functions in normal and PCa samples.

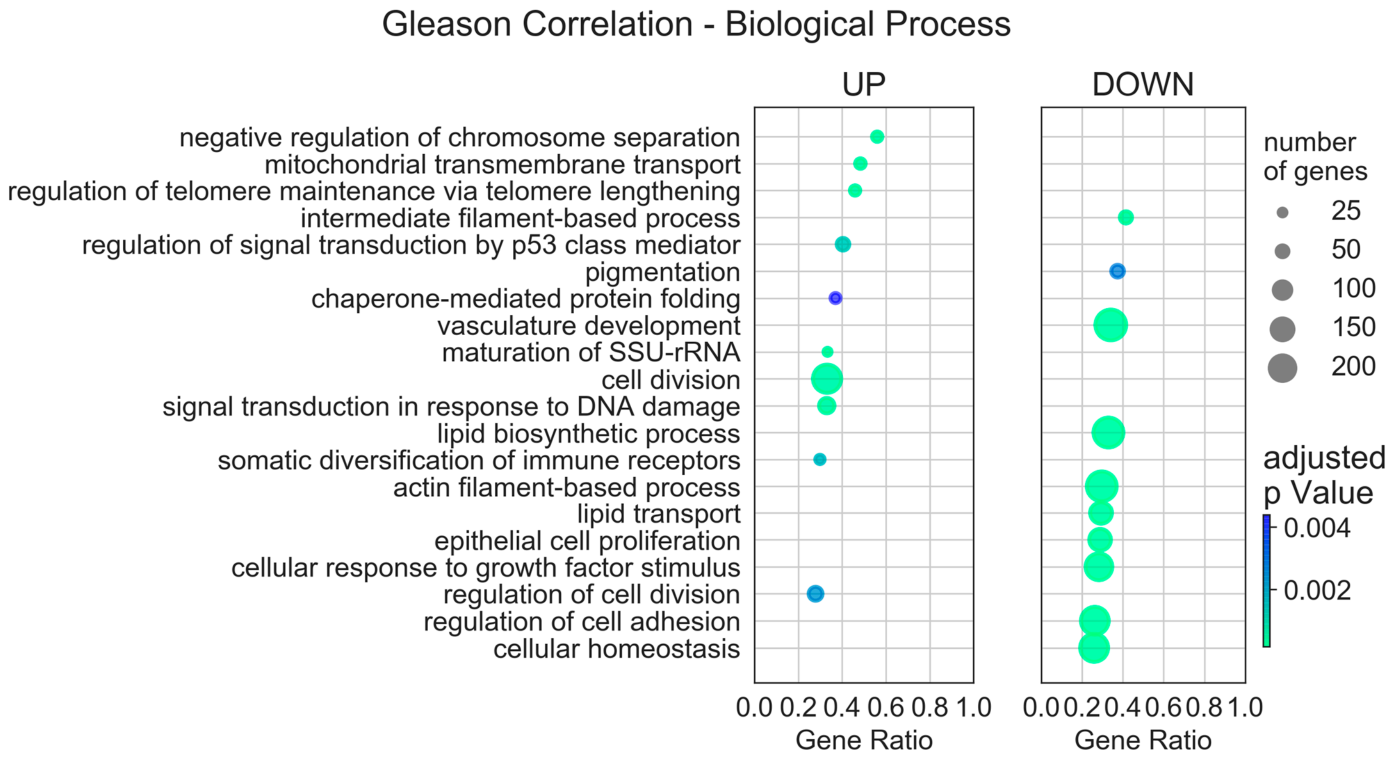


**Figure 2B.** Gene ontology analysis showing upregulated and downregulated set of genes associated with biological processes in Gleason grade groups.

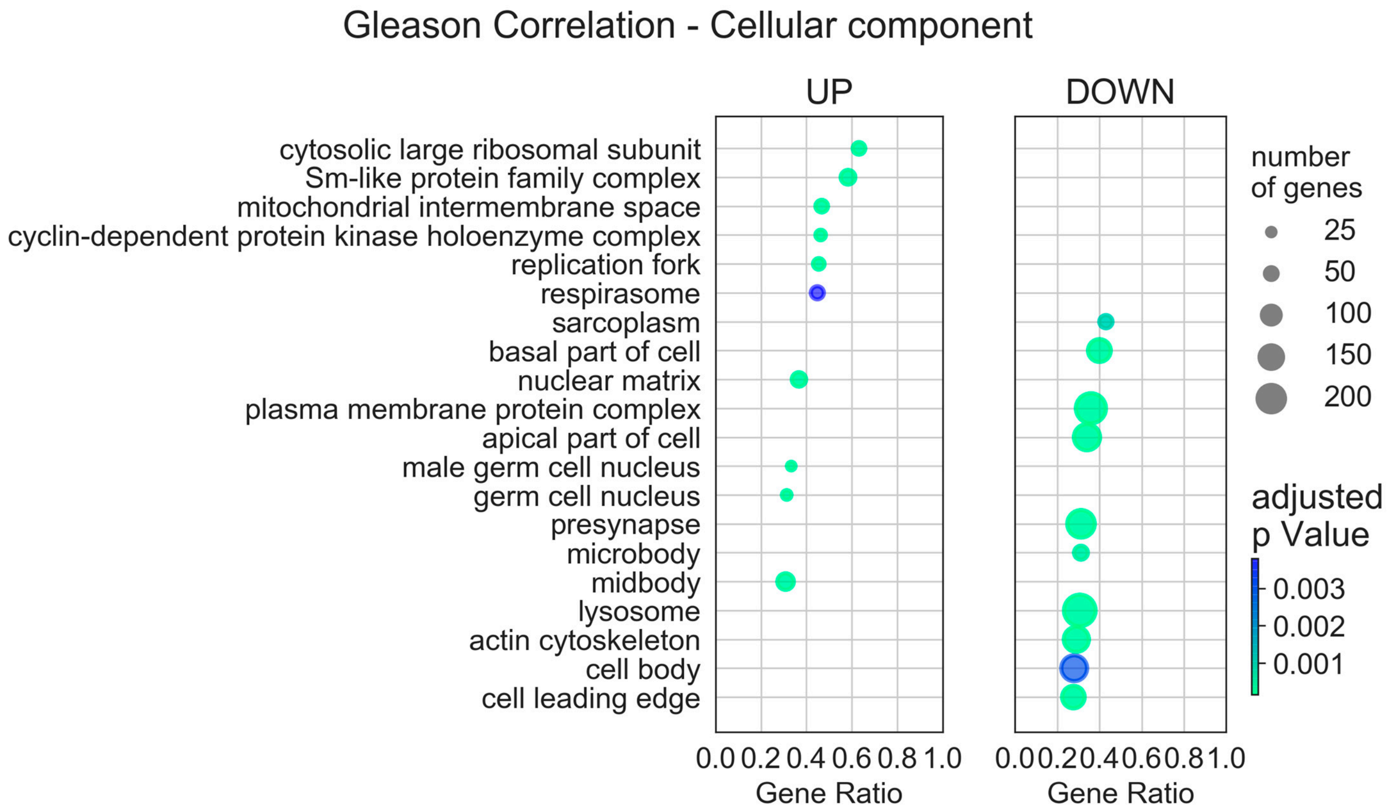

**Figure 2C.** Gene ontology analysis showing upregulated and downregulated set of genes associated with cellular components in Gleason grade groups.

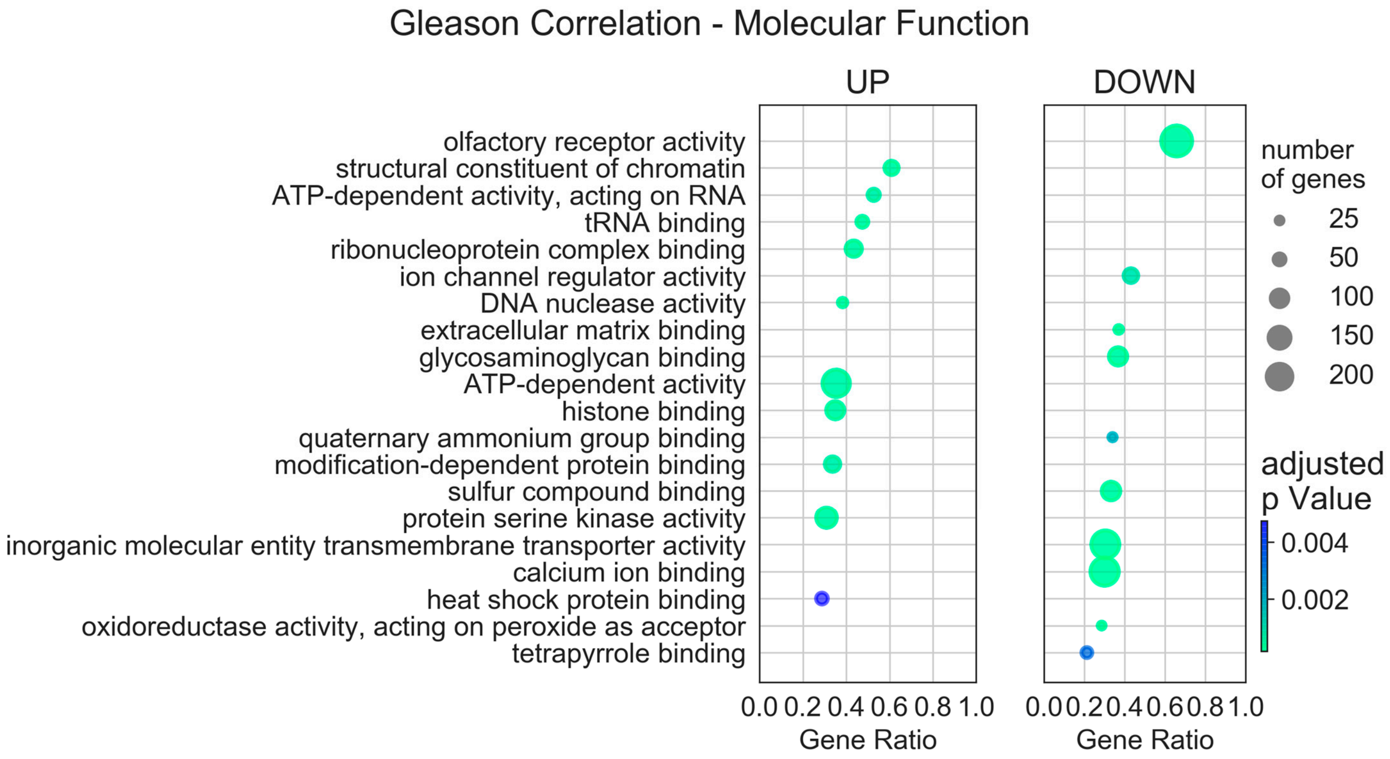


**Figure 2D.** Gene ontology analysis showing upregulated and downregulated set of genes associated with molecular functions in Gleason grade groups.
